# Impact of Hearing Impairment on Missingness of Cognitive Test Scores in the ARIC Study

**DOI:** 10.1093/geroni/igaa057.2995

**Published:** 2020-12-16

**Authors:** Jennifer Deal, Alden Gross, Alison Abraham, A Richey Sharrett, Nicholas Reed, Thomas Mosley, Frank Lin, Bonnielin Swenor

**Affiliations:** 1 Johns Hopkins Bloomberg School of Public Health, Baltimore, Maryland, United States; 2 Johns Hopkins University School of Medicine, Baltimore, Maryland, United States; 3 Johns Hopkins University, Baltimore, Maryland, United States; 4 University of Mississippi Medical Center, Jackson, Mississippi, United States

## Abstract

Despite its high prevalence, the impact of hearing impairment on completion of cognitive tests, many of which rely on auditory input to access test material, has not been described. We investigated if hearing impairment is associated with missing scores in 3602 adults (72-94 years, 23% black, 60% female). Cognition was measured using 10 neurocognitive tests. Pure tone better-ear hearing thresholds (0.5-4 kHz) were averaged and categorized. ≥Moderate hearing impairment (versus none) was associated with greater missingness on two auditory tests: Logical Memory (prevalence ratio [PR]:1.40, 95% confidence interval [CI]:1.01,1.70) and Digits Backwards (PR:1.35, 95% CI:1.00,1.82); and the non-auditory Trail Making Test Part B (PR:1.48, 95% CI:1.24,1.77). Compared to models using complete cognitive data, models that imputed missing scores showed stronger associations of hearing impairment with poor cognitive performance. Older adults with HI are less likely to complete cognitive testing, resulting in biased estimates of the hearing impairment-cognitive performance relationship.

